# Construction of Co_3_O_4_/ZnO Heterojunctions in Hollow N‐Doped Carbon Nanocages as Microreactors for Lithium–Sulfur Full Batteries

**DOI:** 10.1002/advs.202300860

**Published:** 2023-04-20

**Authors:** Biao Wang, Yilun Ren, Yuelei Zhu, Shaowei Chen, Shaozhong Chang, Xiaoya Zhou, Peng Wang, Hao Sun, Xiangkang Meng, Shaochun Tang

**Affiliations:** ^1^ National Laboratory of Solid State Microstructures Collaborative Innovation Center of Advanced Microstructures College of Engineering and Applied Sciences Nanjing University Jiangsu 210093 China; ^2^ Frontiers Science Center for Transformative Molecules School of Chemistry and Chemical Engineering and Zhangjiang Institute for Advanced Study Shanghai Jiao Tong University Shanghai 200240 China

**Keywords:** electrocatalyst, heterojunctions, lithium dendrites, lithium–sulfur batteries, shuttling effect

## Abstract

Lithium–sulfur (Li–S) batteries are promising alternatives of conventional Li‐ion batteries attributed to their remarkable energy densities and high sustainability. However, the practical applications of Li–S batteries are hindered by the shuttling effect of lithium polysulfides (LiPSs) on cathode and the Li dendrite formation on anode, which together leads to inferior rate capability and cycling stability. Here, an advanced N‐doped carbon microreactors embedded with abundant Co_3_O_4_/ZnO heterojunctions (CZO/HNC) are designed as dual‐functional hosts for synergistic optimization of both S cathode and Li metal anode. Electrochemical characterization and theoretical calculations confirm that CZO/HNC exhibits an optimized band structure that effectively facilitates ion diffusion and promotes bidirectional LiPSs conversion. In addition, the lithiophilic nitrogen dopants and Co3O4/ZnO sites together regulate dendrite‐free Li deposition. The S@CZO/HNC cathode exhibits excellent cycling stability at 2 C with only 0.039% capacity fading per cycle over 1400 cycles, and the symmetrical Li@CZO/HNC cell enables stable Li plating/striping behavior for 400 h. Remarkably, Li‐S full cell using CZO/HNC as both cathode and anode hosts shows an impressive cycle life of over 1000 cycles. This work provides an exemplification of designing high‐performance heterojunctions for simultaneous protection of two electrodes, and will inspire the applications of practical Li–S batteries.

## Graphical abstract

An advanced microreactor embedded with Co_3_O_4_/ZnO heterojunctions (CZO/HNC) is designed as dual‐functional hosts for synergistic optimization of both S cathode and Li anode. Electrochemical characterization and theoretical calculations confirm that CZO/HNC exhibits an optimized band structure that effectively facilitates ion diffusion and promotes polysulfides conversion. In addition, the lithiophilic nitrogen dopants and Co_3_O_4_/ZnO sites together regulate dendrite‐free Li deposition.

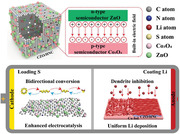

## 1 Introduction

The rapid development of electric vehicles and portable electronics has aroused the enthusiasm of exploring new battery systems with energy densities higher than 400 Wh kg^−1^.^[^ ^1^ ^]^ Lithium–sulfur (Li–S) batteries demonstrate a bright commercialization prospect because of their appealing advantages, such as the ultra‐high theoretical energy density (2600 Wh g^−1^), low reduction potential of Li metal (−3.04 V versus standard hydrogen electrode), and natural abundance.^[^ ^2^ ^]^ Unfortunately, there are still some critical barriers that impede the commercialization of Li–S batteries. In terms of the S cathode, for instance, the serious shuttle effect of lithium polysulfides (LiPSs), sluggish electrochemical kinetics, and huge volume expansion result in poor electrochemical performances.^[^ ^3^ ^]^ As for the Li metal anode, the nonuniform Li deposition leads to the formation of dead Li or even Li dendrites, which leads to inferior safety.^[^ ^4^ ^]^

In light of the aforementioned issues, great strategies have been made to construct advanced electrodes. To solve the obstacles on the S cathode, various polar catalytic materials with well‐designed structure were applied to immobilize LiPSs and promote the redox reaction between S and Li_2_S.^[^ ^5^ ^]^ In order to build stable Li anode, considerable approaches such as constructing artificial interface layer, introducing electrolyte additives, and designing lithiophilic hosts have been developed.^[^ ^6^ ^]^ Typically, transition‐metal oxides (TMOs) with low cost and strong polarity have been demonstrated to be attritive S or Li host, which can be regarded as “seeds” to adsorb LiPSs on cathode and induce the deposition behavior of Li on anode.^[^ ^7^ ^]^ However, their practical performance is much lower than the expectations due to their poor conductivity, weak catalytic activity, and inferior stability. It is demonstrated that heterojunction between nanocrystals having different band structures can induce an internal electric field between their heterointerface to facilitate electron/ion transport and improve the surface reaction kinetics.^[^ ^8^ ^]^ Furthermore, the heterojunctions offer a strong synergistic enhancement by all individual components to provide comprehensive improvement in terms of factors such as electron transfer, redox kinetics, and LiPSs adsorption in a Li–S full cell. In addition, it was proposed that the lager interiors void of hollow carbon materials, especially heteroatom doped carbons, can not only provide more exposed surfaces to anchor TMO, but also play an indispensable role to maintain the structural stability during cycling process.^[^ ^9^ ^]^ On accounts of above discussions, combining the advantages of TMO heterojunctions and hollow heteroatom doped carbons through straightforward structural design and electronic engineering is important for constructing high performance Li–S full batteries, but there are few relevant reports.

Herein, we show an advanced dual‐functional hosts comprised of Co_3_O_4_/ZnO heterojunctions encapsulated in hollow N‐doped carbon nanocages (CZO/HNC) to stabilize both the S cathode and Li metal anode. The built‐in electric field generated between the phase boundaries from the ZnO to Co_3_O_4_ can promote the interfacial charge transfer and reaction kinetics. In addition, the CZO/HNC provides sufficient active sites for adsorption of LiPSs on cathode and induction of homogeneous Li nucleation on anode. The detailed mechanism has been further verified by density functional theory (DFT) calculations, in situ Raman spectrum, and electrochemical performance. As a result, full protection of the two electrodes of the coupled Li–S full cell leads to remarkable long cycling life over 1000 cycles at 0.5 °C with an ultralow decay rate of 0.05% per cycle. This study deepens understanding of designing high‐performance heterojunctions for accelerating polysulfides redox kinetics and inhibiting lithium dendrites.

## 2 Results and Discussion

**Figure 1:**
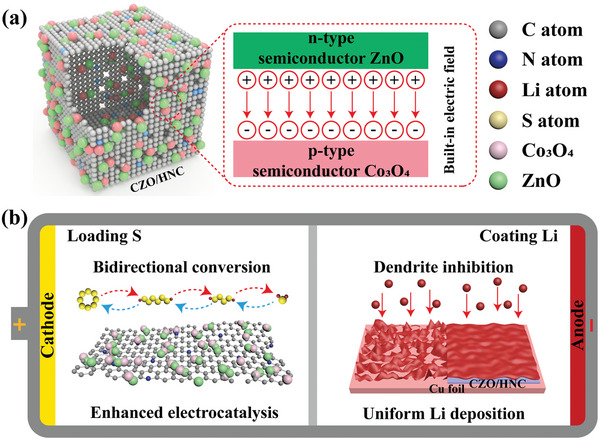
Schematic illustration of a) Co_3_O_4_/ZnO heterojunctions in CZO/HNC, and b) the role played by CZO/HNC in Li–S full cell.

The porous hollow N‐doped carbon nanocages embedded with Co_3_O_4_/ZnO heterojunctions were fabricated by in situ thermal treatment of Co/Zn MOFs in air (see details in Supporting Information). Because of the p‐type and n‐type semiconductor characteristics of Co_3_O_4_ and ZnO, respectively, the different energy structures can induce an interfacial polarization, which leads to oriented charge separation and results in built‐in electric field (BIEF) at the interface, as shown in **Figure** **1**a. Clearly, the formation of BIEF can change charge density around the Co_3_O_4_ and ZnO to enhance electron transfer and reactant diffusion, thus improving the redox kinetics and the overall reactivity.^[^ ^4^, ^10^ ^]^ As cathode host, the BIEF of Co_3_O_4_/ZnO heterojunctions ensures durable and efficient conversion of LiPSs by accelerating the charge transfer. As anode host, our CZO/HNC is beneficial to afford abundant active sites for sufficient Li nucleation and deposition. Therefore, both the shuttle effect and Li dendrite growth can be effectively suppressed based on our CZO/HNC host (Figure 1b).

**Figure 2:**
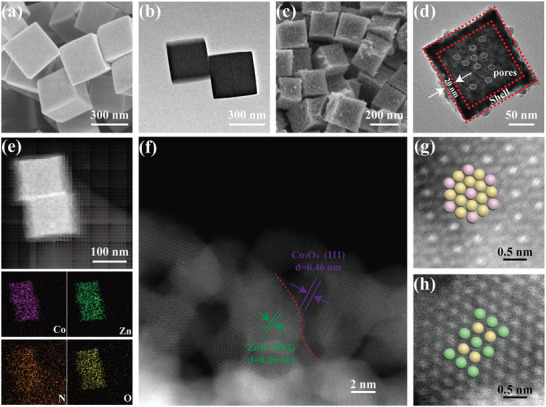
a,c) SEM and b,d) TEM images of Co/Zn MOFs and CZO/HNC. e) HAADF‐STEM image and the corresponding EDS elemental maps of CZO/HNC. f–h) The spherical‐aberration‐corrected HAADF‐STEM images of CZO/HNC.

The scanning electron microscope (SEM) and transmission electron microscopy (TEM) images of Co/Zn MOFs showed that these precursors have a well‐defined cube‐like morphology and smooth surfaces with an average edge length of roughly 350 nm (**Figure** **2**a,b). After a simple calcination, the resulting CZO/HNC turned into hollow structured nanocage, and the porous wall thickness of cage is about 20 nm (Figure 2c,d). Energy dispersive spectroscopy (EDS) result (Figure S1, Supporting Information) revealed that the atomic percentages of C, N, Co, Zn, and O elements in the CZO/HNC are about 18.3, 0.7, 20.1, 19.3, and 41.6 at. %, respectively. The generation of hollow nanostructures is the result of nanoscale Kirkendall effect.^[^ ^11^ ^]^ Typically, Co/Zn MOFs were exposed to air under elevated temperatures in muffle furnace. When the outward diffusion rate of the metal cations is much higher than the inward diffusion rate of the anions, the vacancy diffusion occurred to compensate the diffusivity difference. Excessive inward flux of vacancies will merge into a void. The inner hollow cavities provided a high specific surface area of the CZO/HNC, which could accommodate the volume expansion of S and promote the transfer of Li‐ion by improving the wettability of electrolyte.^[^ ^12^ ^]^ High‐angle annular dark‐field scanning TEM (HAADF‐STEM) image and the corresponding EDS mappings (Figure 2e) elucidated the homogeneous distribution of Co, Zn, N, and O elements in the CZO/HNC. The spherical aberration‐corrected HAADF‐STEM image of CZO/HNC further revealed the heterointerfaces between Co_3_O_4_ and ZnO, where the measured lattice spacings of 0.46 and 0.26 nm associate with the Co_3_O_4_ (111) plane and the ZnO (002) plane, respectively.^[^ ^9^, ^13^ ^]^ Their tightly contacted interfaces can be regarded as active sites rapid diffusion of LiPSs from Co_3_O_4_ to ZnO. The atomic distribution models were shown in the Figure 1g,h, in which the red, green, and yellow balls represent Co, Zn, and O atoms, respectively.^[^ ^14^ ^]^ For comparison, Co_3_O_4_/HNC and ZnO/HNC were also prepared using Co MOFs and Zn MOFs under similar conditions (Figures S2–S4, Supporting Information).

The X‐ray diffraction (XRD) patterns (Figure S5, Supporting Information) showed distinct peaks corresponding to Co_3_O_4_ (PDF#42‐1467) and ZnO (PDF#36‐1451) phases, which indicates the co‐existence of Co_3_O_4_ and ZnO in the CZO/HNC. Furthermore, X‐ray photoelectron spectroscopy (XPS) spectra were conducted to investigate the interaction between Co_3_O_4_ and ZnO (Figure S6, Supporting Information). As shown in Figure S7 (Supporting Information), the peaks of Co and Zn of CZO/HNC shifted toward lower and higher binding energy compared to Co_3_O_4_/HNC and ZnO/HNC, respectively. This might be attributed to electron transfer from ZnO to Co_3_O_4_, which is favorable to the stable construction of heterointerfaces with a fast charge transfer channel.^[^ ^15^ ^]^ In addition, the decrease of Co^3+^/Co^2+^ atomic ratio also proved reduction of the Co oxidation state. Moreover, the peak located at 531.1 eV in the high‐resolution O 1*s* spectrum of CZO/HNC (Figure S8, Supporting Information) displayed the presence of oxygen vacancies, which could improve the intrinsic conductivity of the CZO/HNC.^[^ ^10^, ^16^ ^]^ The specific surface area and pore volume were performed by N_2_ adsorption‐desorption measurement and the results were shown in Figure S9 and Table S1 (Supporting Information). The hierarchical mesopores provided excellent adsorption of electrolyte to enable the sufficient wetting of active sites of heterojunction.^[^ ^12^ ^]^

**Figure 3:**
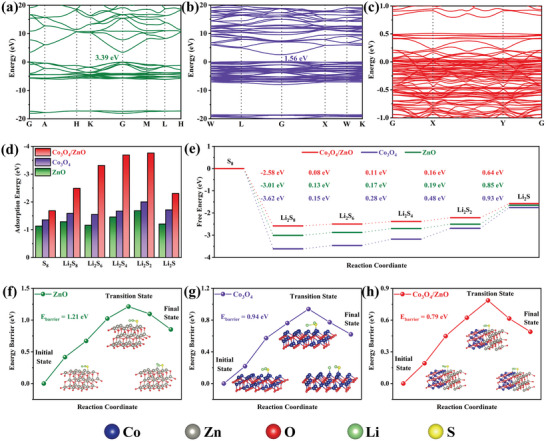
Band structures of a) ZnO, b) Co_3_O_4_, and c) Co_3_O_4_/ZnO. d) Calculated adsorption energy of sulfur species with ZnO, Co_3_O_4_, and Co_3_O_4_/ZnO heterointerface. e) Gibbs free energy of LiPSs on ZnO, Co_3_O_4_, and Co_3_O_4_/ZnO heterointerface. Energy profiles of the decomposition barriers of Li_2_S on f) ZnO, g) Co_3_O_4_, and h) Co_3_O_4_/ZnO heterointerface (insets: the initial, transition, and final structures).

We performed DFT calculations to investigate the adsorption and electrocatalysis capabilities of different structures for LiPSs. Three models corresponding to ZnO, Co_3_O_4_, and Co_3_O_4_/ZnO heterojunction were considered in the DFT simulations (Figure S10, Supporting Information). Band structures (**Figure** **3**a,b) displayed that Co_3_O_4_ and ZnO have a wide energy bandgap of 1.56 and 3.39 eV respectively, indicating low electronic conductivity. After coupling into heterojunction, the Co_3_O_4_/ZnO heterointerface possessed a negligible energy bandgap (Figure 3c). To better understand the electronic properties of Co_3_O_4_/ZnO, the total density of states (TDOS) of ZnO, Co_3_O_4_, and Co_3_O_4_/ZnO were calculated. According to Figure S11 (Supporting Information), the TDOS of Co_3_O_4_/ZnO have no gap around the Fermi level.^[^ ^7^, ^17^ ^]^ In addition, the electrical conductivities of the Co_3_O_4_/HNC, ZnO/HNC, and CZO/HNC were measured by four‐point probes resistivity system to be 1.7×10^−3^, 3.2×10^−4^ and 6.6×10^−3^ S m^−1^, respectively. This further proves that the conductivity of Co_3_O_4_/ZnO heterojunctions is higher than that of Co_3_O_4_ and ZnO.

The optimized configurations were presented in Figure S12‐S14, from which the calculated results showed that the adsorption energies of sulfur species on the Co_3_O_4_/ZnO were all lower than those on Co_3_O_4_, and ZnO (Figure 3d). This was further confirmed by visual adsorption experiments showing time‐dependent color change of Li_2_S_6_ solutions containing various adsorbents (Figure S15). After adding the CZO/HNC and being kept for 6 h, the Li_2_S_6_ solution completely turned from yellowish into transparentness, indicating that Co_3_O_4_/ZnO heterojunctions can efficiently mitigate the shuttling of LiPSs. As shown in Figure 3e, the reaction free‐energies for the discharging process from S_8_ to Li_2_S were calculated to evaluate the electrochemical reaction kinetics. Clearly, the reaction from S_8_ to Li_2_S_8_ was spontaneous exothermic. Meanwhile, the free‐energy from Li_2_S_2_ to Li_2_S was the largest in these unspontaneous reactions, indicating the reaction was a rate‐limited step during the discharge process.^[^ ^18^ ^]^ The free energy of rate‐limited step was 0.64 eV for Co_3_O_4_/ZnO while 0.93 eV for Co_3_O_4_ and 0.85 eV for ZnO, indicating that the reduction of S was thermodynamically more favorable on the Co_3_O_4_/ZnO heterojunctions than that on single component.

It is well known that the reduction products tend to deposit on the surface of the host, which is detrimental to the catalytic ability of the material. Therefore, it is necessary to simultaneously focus on the rapid transformation (reduction ability) and generation (oxidation ability) of LiPSs. In terms of the Li_2_S dissociation process, the calculated decomposition barrier of Co_3_O_4_/ZnO heterointerface with adsorbed Li_2_S (0.79 eV) was smaller than Co_3_O_4_ (0.94 eV) and ZnO (1.21 eV), suggesting that Co_3_O_4_/ZnO accelerates the phase transformation of Li_2_S.^[^ ^19^ ^]^ Overall, DFT results demonstrated the enhancement of Li_2_S nucleation and decomposition kinetics on the Co_3_O_4_/ZnO heterointerface at the atomic level, which further indicates that the heterostructure is an efficient bidirectional electrocatalyst in Li–S batteries.

**Figure 4:**
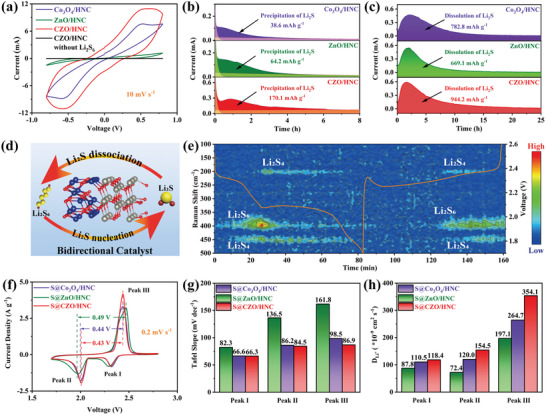
a) CV profiles of symmetrical cells at 10 mV s^−1^. b) Potentiostatic discharge and c) potentiostatic charge profiles of Co_3_O_4_/HNC, ZnO/HNC, and CZO/HNC electrodes. d) Schematic illustration of one‐directional catalyst with heterostructure Co_3_O_4_/ZnO. e) In situ Raman spectra with S@CZO/HNC cathode during the first cycle at 0.5 C. f) CV profiles of different cathodes at 0.2 mV s^−1^. g) Tafel slopes and h) Li‐ion diffusion coefficient values at peaks I, II, and III.

Cyclic voltametric (CV) of symmetrical cells were performed using the Li_2_S_6_ electrolyte to explore the electrocatalytic activity of different catalysts. As shown in **Figure** **4**a, symmetrical CZO/HNC cell delivered a much larger redox current than those for the Co_3_O_4_/HNC and ZnO/HNC cells, attributing to the strong catalytic effect of CZO/HNC.^[^ ^20^ ^]^ In addition, the rapid reaction kinetics can also be identified by the electrochemical impedance spectroscopy (EIS) analysis since these symmetric cells exclude the influence of Li foil.^[^ ^21^ ^]^ As shown in Figure S16 (Supporting Information), the symmetrical cells displayed a semicircle in the high‐frequency region, which is attributed to the charge transfer resistance (*R*_ct_). The *R*_ct_ value for symmetrical CZO/HNC cell is only 37.2 Ω, which is much lower than that of Co_3_O_4_/HNC cell (69.7 Ω) and ZnO/HNC cell (171.8 Ω). This demonstrates that the CZO/HNC heterostructure greatly accelerates charge transfer to contribute the redox kinetic of LiPSs conversion. The potentiostatic discharge and potentiostatic charge tests of Co_3_O_4_/HNC, ZnO/HNC, and CZO/HNC electrodes were used to investigate the enhanced bidirectional S conversion by the Co_3_O_4_/ZnO.^[^ ^22^ ^]^ As shown in Figure 4b, the Li_2_S deposition capacity of CZO/HNC (170.1 mAh g^−1^) was higher than that of Co_3_O_4_/HNC (38.6 mAh g^−1^) and ZnO/HNC (64.2 mAh g^−1^), indicating that the Co_3_O_4_/ZnO heterojunction greatly promotes Li_2_S nucleation and growth. To reveal the influence of different host on Li_2_S deposition, the Li_2_S deposition morphology was observed by SEM. It is obvious that uniform Li_2_S deposition was observed on carbon paper‐CZO/HNC, whereas Li_2_S aggregation clearly occurred on the surfaces of carbon paper‐Co_3_O_4_/HNC and carbon paper‐ZnO/HNC (Figure S17, Supporting Information).^[^ ^23^ ^]^ For the Li_2_S dissolution process, CZO/HNC displayed a much higher current peak and earlier dissolution of Li_2_S compared to the other two cells (Figure 4c). These results demonstrated that Co_3_O_4_/ZnO heterojunctions boost bidirectional S conversion, as schematically shown in Figure 4d. In addition, in situ Raman spectroscopy was used to monitor the conversions of various LiPSs during discharge/charge processes. As shown in Figure 4e, the intensities of S_6_^2−^ peaks (400 and 450 cm^−1^) gradually declined as the discharge process, followed by regeneration upon charging, which demonstrates the efficient and reversible conversion of LiPSs in S@CZO/HNC cell.^[^ ^24^ ^]^

The CV profiles of S@CZO/HNC cathode for the first two cycles at 0.1 mV s^−1^ are shown in Figure S18 (Supporting Information). Two cathodic peaks located at ≈2.32 and 2.04 V were assigned to transformation of S_8_ to LiPSs and then to Li_2_S, while the appearance of one anodic peak at ≈2.43 V can be attributed to the conversion of LiPSs to S_8_.^[^ ^25^ ^]^ The voltage gap between peak II and peak III in Figure 4f represents polarization potential in the redox reaction and the peak current reflects the redox kinetics.^[^ ^15a^ ^]^ Compared with S@Co_3_O_4_/HNC and S@ZnO/HNC cells, S@CZO/HNC cell showed much smaller polarization and higher peak current, indicating that Co_3_O_4_/ZnO heterojunctions can profitably promote the electrochemical reactions. To further investigate the catalytic influence of different hosts, Tafel plots of three peaks are shown in Figure 4g and Figure S19 (Supporting Information). S@CZO/HNC cell showed the lowest Tafel slope for the reduction and the oxidation reactions, indicating that CZO/HNC simultaneously accelerates the conversion of LiPSs and Li_2_S. In addition, CV curves of the three cells from 0.2 to 0.5 mV s^−1^ were carried out (Figure S20, Supporting Information). As shown in Figure S21 (Supporting Information), the peak currents vary linearly with the square root of the scan rate. According to Randles–Sevcik equation, the diffusion capacity of Li‐ion can be evaluated by the slope of the line.^[^ ^26^ ^]^ As summarized in Figure 4h, all slopes of S@CZO/HNC cell had the largest values, indicating much faster Li‐ion diffusivity in whole discharge/charge process. Besides, the S@CZO/HNC cell also delivered a lower *R*_ct_ (62.2 Ω) compared to S@Co_3_O_4_/HNC cell (102.6 Ω) and S@ZnO/HNC cell (115.0 Ω), indicating enhanced charge transfer at the electrode/electrolyte interface (Figure S22, Supporting Information).

**Figure 5:**
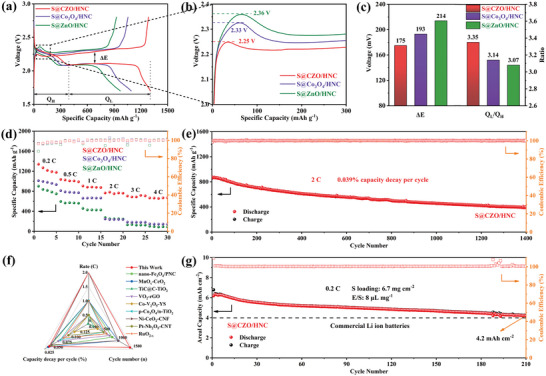
a,b) GCD voltage curves at 0.2 C c) The values of Δ*E* and Q_L_/Q_H_ obtained from discharge/charge voltage curves. d) Rate capability of different cathodes. e) Cycling performance of S@CZO/HNC at 2 C. f) Cell performance comparison with previously reported Li–S cells. g) Cycling performance of S@CZO/HNC cathode with 6.7 mg cm^−2^ S loading at 0.2 C.

Charge–discharge behaviors were investigated by using S@Co_3_O_4_/HNC, S@ZnO/HNC, and S@CZO/HNC cathodes (≈75 wt.% S, Figure S23, Supporting Information). At 0.2 C (1 C = 1675 mA g^−1^), all the cathodes showed the curve shape of typical Li–S batteries, demonstrating one charging and two discharging plateaus that is consistent with the CV profile (**Figure** **5**a). Notably, the lower overpotential of S@CZO/HNC cell indicates the outstanding kinetics (Figure 5b).^[^ ^23a^ ^]^ In addition, a voltage gap Δ*E* (the voltage gap at 50% discharged capacity) is observed between the oxidation and the second reduction plateaus to reflect the redox kinetics.^[^ ^27^ ^]^ The S@CZO/HNC cell displayed a lower polarization potential than S@Co_3_O_4_/HNC and S@ZnO/HNC cells, as consequence of the superior electrocatalytic activity of Co_3_O_4_/ZnO heterojunctions towards LiPSs conversion. On the other hand, LiPSs cannot completely convert into Li_2_S due to the sluggish reaction kinetics during the second plateau (Q_L_), which inhibits the release of capacity during the Q_L_ stages. Thus, the catalytic ability of the host toward the LiPSs conversion reaction can be quantified by the ratio of Q_L_/Q_H_. S@CZO/HNC cell exhibited a larger Q_L_/Q_H_ value than those of S@Co_3_O_4_/HNC and S@ZnO/HNC cells (Figure 5c), implying the best catalytic activity.^[^ ^18a^ ^]^

The efficient S utilization of S@CZO/HNC enabled a high specific capacity of 1306 mAh g^−1^ at 0.2 C (Figure S24, Supporting Information), which is superior to S@Co_3_O_4_/HNC (1092.1 mAh g^−1^) and S@ZnO/HNC (970.3 mAh g^−1^). The rate capability of the S@CZO/HNC, S@Co_3_O_4_/HNC, and S@ZnO/HNC cells displayed that S@CZO/HNC showed the best rate capability compared to the other two counterparts (Figure 5d). At a higher rate, e.g., 4 C, S@CZO/HNC showed a discharge capacity of 665.7 mAh g^−1^, and corresponding capacity of S@Co_3_O_4_/HNC and S@ZnO/HNC cells are 148.0 and 208.2 mAh g^−1^. The polarization degree of Li–S battery enhances with increasing C‐rate, as demonstrated by galvanostatic charge/discharge (GCD) voltage curves (Figure S25, Supporting Information). Due to enhanced reaction kinetics in S@CZO/HNC cell, two typical discharge plateaus are maintained even at a high rate of 4 C. The S@CZO/HNC cell also demonstrated an ultralow capacity fade of 0.039% per cycle during 1400 cycles at 2 C (Figure 5e), which represented one of the highest cycling stabilities among state‐of‐the‐art Li–S batteries (Figure 5f; Table S2, Supporting Information). Impressively, with a high S loading of 6.7 mg cm^−2^, the S@CZO/HNC cell could maintain an aerial capacity of 4.2 mAh cm^−2^ after 210 cycles at 0.2 C (Figure 5g), suggesting that CZO/HNC can serve as an effective cathode host that inhibits the shuttle effect in practical Li–S cells.

**Figure 6:**
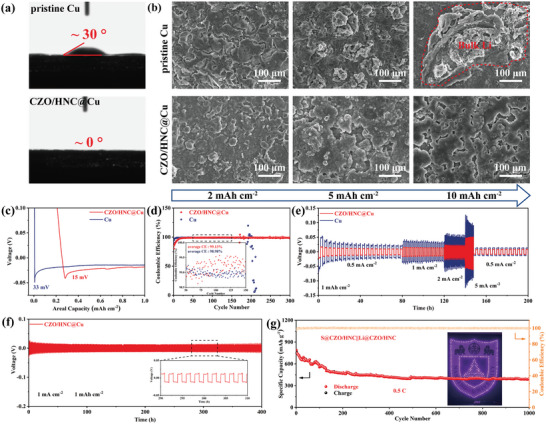
a) Comparison of the contact angles of electrolyte on pristine Cu and CZO/HNC@Cu substrates. b) SEM images of pristine Cu and CZO/HNC@Cu hosts after plating with various deposition capacities. c)Voltage‐capacity profiles and d) CE of the Li plating‐stripping progress on pristine Cu and CZO/HNC@Cu hosts. e) Rate performance at current densities from 0.5 to 5 mA cm^−2^. f) Cycling performance of the symmetrical cells using Li@CZO/HNC@Cu. g) Long cycling performance of the S@CZO/HNC||Li@CZO/HNC full cell. The inset is a digital image showing LEDs powered by our Li–S cull cell.

The electrolyte wettability is one of the key factors for reversible Li stripping/plating, which affects the interfacial ion transport resistance.^[^ ^28^ ^]^ In the contact angle measurement, CZO/HNC@Cu possesses significantly enhanced wettability to electrolyte, showing a contact angle of nearly 0° that is much better than 30° based on pristine Cu foil (**Figure** **6**a). The morphology of Li deposition on CZO/HNC@Cu and Cu anodes were observed with different areal capacities. For a bare Cu foil, Li deposition with obviously larger particles can be observed at a plating capacity of 5 mAh cm^−2^ (Figure 6b). With a higher areal capacity of 10 mAh cm^−2^, bulk Li protrusions cover almost the whole host, which will puncture the separator. In presence of the CZO/HNC layer, homogenous Li deposition was observed on the surface of CZO/HNC@Cu. As shown in Figure 6c, a much smaller Li nucleation overpotential of 15 mV was endowed to the CZO/HNC@Cu cell (33 mV for Cu cell) when Li metal were used as counter electrodes, indicating the favorable lithiophilicity of CZO/HNC with enhanced Li nucleation.^[^ ^29^ ^]^ In the Li deposition process, Li first reacts with Co_3_O_4_/ZnO to generate lithiophilic Co and Zn atoms.^[^ ^4^, ^9^ ^]^ The corresponding reactions are as follows:

(1) $${\mathrm{Li}}^{+}+{\mathrm{Co}}_{3}O_{4}\to {\mathrm{Li}}_{2}O+\mathrm{Co}$$

(2) $${\mathrm{Li}}^{+}+\mathrm{ZnO}\to {\mathrm{Li}}_{2}O+\mathrm{Zn}$$

The lithiophilic Co and Zn atoms can effectively control the nucleation and growth behavior of Li to achieve the flat and uniform deposition. Meanwhile, the pyridinic‐N and pyrrolic‐N doped carbons provide stable Li adsorption sites. The adsorbed Li atoms are located above or in the vacancy of the defects in pyridinic‐N and pyrrolic‐N doped carbons.^[^ ^7^, ^9^ ^]^ Therefore, the enhanced lithiophilicity of CZO/HNC is attributed to the synergistic effect of the nitrogen dopants and Co_3_O_4_/ZnO sites.

After that, the CZO/HNC@Cu cell displayed excellent reversibility over 300 cycles (Figure 6d). In contrast, the Coulombic efficiency (CE) of Cu cell showed a sharp fluctuation after 180 cycles, which was caused by the uncontrollable Li dendrite formation on the surface of the Cu foil. The average CE of CZO/HNC@Cu cell (99.13%) is higher than that of Cu cell (98.98%), indicating that the CZO/HNC can serve as an efficient Li host with excellent plating/striping stability and reversibility.^[^ ^9b^ ^]^ The rate performances of the Li@CZO/HNC@Cu and Li@Cu symmetrical cells were also examined at various current densities from 0.5 to 5 mA cm^−2^. Clearly, Li@CZO/HNC@Cu showed a lower overpotential at all rates (Figure 6e). Figure 6f and Figure S26 (Supporting Information) confirm the superiority of CZO/HNC in enhancement of cycling stability. The Li@CZO/HNC@Cu symmetrical cell exhibits an outstanding cycling stability over 400 h with a highly stable overpotential (15 mV) at 1 mA cm^−2^. These results suggested that the stable Li stripping/plating behavior induced by CZO/HNC possessed great potentials to achieve high‐performing Li–S full cells.

We further evaluated the electrochemical performances of S@CZO/HNC||Li@CZO/HNC full cells to prove the feasibility of CZO/HNC as dual‐functional hosts for protection of both cathode and anode. The S@CZO/HNC||Li@CZO/HNC full cell demonstrated excellent rate capability from 0.2 to 2 C, delivering a remarkable specific capacity of 507.0 mAh g^−1^ even at 2 C (Figure S27, Supporting Information). It further showed an ultralow decay rate of 0.05% per cycle after 1000 cycles at 0.5 C with sulfur loading of 2 mg cm^−2^, which still kept a stable cycling trend (Figure 6g). The excellent electrochemical performances demonstrated that our CZO/HNC could satisfy the high‐efficiency protection of the S cathode and Li metal anode simultaneously. As a proof of concept, we used the two Li–S cells to power a LED array panel (inset of Figure 6g).

## 3 Conclusions

In conclusion, the unique porous hollow N‐doped carbon nanocages embedded with Co_3_O_4_/ZnO heterojunctions were proposed and constructed to address the problems of Li anode and S cathode at the same time. In this design, the hollow carbon nanocages provide stable frameworks for exposing active surfaces to improve catalytic/adsorptive performance and physically limiting the dissolution of LiPSs. In addition, the Co_3_O_4_/ZnO heterojunction with optimized energy band structure is not only beneficial for the adsorption and conversion of LiPSs, but also favorable for the homogeneous deposition of Li metal. Benefiting from these synergistic effects, the suppression of shuttle effect and lithium dendrite are realized in one battery. Therefore, the Li–S full batteries with CZO/HNC exhibit good rate capacity of 507.0 mAh g^−1^ at 2 C with 3.8 mg cm^−2^ S loading and stable cycling over 1000 cycles with a low decay rate of 0.05% per cycle at 0.5 C. It is believed that the rational design of bifunctional hosts will promote the commercialization of Li–S batteries.

## Conflict of Interest

The authors declare no conflict of interest.

## Supporting information

Supporting Information

## Data Availability

The data that support the findings of this study are available from the corresponding author upon reasonable request.
