# Supplementary material for: Construction of Co3O4/ZnO Heterojunctions in Hollow N‐Doped Carbon Nanocages as Microreactors for Lithium–Sulfur Full Batteries
Source: Adv Sci (Weinh). 2023 Apr 20;10(19):2300860. doi: 10.1002/advs.202300860 (PMC10323615; doi:10.1002/advs.202300860)
Supplement: Supplementary file 1 — Supporting Information [file ADVS-10-2300860-s001.pdf]

ADVANCED SCIENCE
Open Access

Supporting Information

for *Adv. Sci.*, DOI 10.1002/advs.202300860

Construction of Co_3O_4/ZnO Heterojunctions in Hollow N-Doped Carbon Nanocages as Microreactors for Lithium–Sulfur Full Batteries

Biao Wang, Yilun Ren, Yuelei Zhu, Shaowei Chen, Shaozhong Chang, Xiaoya Zhou, Peng Wang, Hao Sun, Xiangkang Meng* and Shaochun Tang**

Construction of Co_3O_4/ZnO Heterojunctions in Hollow N-Doped Carbon Nanocages as Microreactors for Lithium-Sulfur Full Batteries

Biao Wang[a=], Yilun Ren[a=], Yuelei Zhu[a=], Shaowei Chen[a], Shaozhong Chang[a], Xiaoya Zhou[a], Peng Wang[a], Hao Sun[b], Xiangkang Meng[a*], Shaochun Tang[a*]*

[a] *National Laboratory of Solid State Microstructures, Collaborative Innovation Center of Advanced Microstructures, College of Engineering and Applied Sciences, Nanjing University, Jiangsu, 210093, China.*

[b] *Frontiers Science Center for Transformative Molecules, School of Chemistry and Chemical Engineering, and Zhangjiang Institute for Advanced Study, Shanghai Jiao Tong University, Shanghai 200240, China.*

Emails: haosun@sjtu.edu.cn; mengxk@nju.edu.cn; tangsc@nju.edu.cn.

Biao Wang, Yilun Ren, and Yuelei Zhu contributed equally to this work.

Experimental section

Synthesis of Co/Zn MOFs, Co MOFs, and Zn MOFs:

For the synthesis of Co/Zn MOFs, 1 mmol $Co(NO_3)_2{\cdot}6H_2O$, 1 mmol $Zn(NO_3)_2{\cdot}6H_2O$, and 30 mg cetyltrimethylammonium bromide (CTAB) were dissolved in 10 mL deionized (DI) water (marked as A). Simultaneously, 9.08 g 2-methyimidazole (2-MIM) were dissolved in another 280 mL DI water (marked as B). Then, A was mixed with B, and stirred for 1.5 h.

Finally, the products were collected by centrifugation, washing, and drying process. For the synthesis of Co MOFs and Zn MOFs, the process is the same as that of Co/Zn MOFs except for using 2 mmol of $Co(NO_3)_2{\cdot}6H_2O$ or $Zn(NO_3)_2{\cdot}6H_2O$ and stirred for 40 min or 6 h respectively.

Synthesis of CZO/HNC, Co_3O_4/HNC, and ZnO/HNC:

The as-prepared Co/Zn MOFs, Co MOFs and Zn MOFs were separately treated at 370 °C for 2 h in air with a heating rate of 0.5 °C min^{-1}. After cooling to room temperature, the obtained materials were labelled as CZO/HNC, Co_3O_4/HNC, and ZnO/HNC respectively.

Synthesis of S@CZO/HNC, S@Co_3O_4/HNC, and S@ZnO/HNC:

Typically, 75% of sulfur and 25% of our prepared materials (CZO/HNC, Co_3O_4/HNC, and ZnO/HNC) were ground uniformly. Then, the mixed powder was transferred into a vacuum-sealed vessel, and treated at 155 °C for 16 h for synthesis of S@CZO/HNC, S@Co_3O_4/HNC, and S@ZnO/HNC.

Materials characterizations:

The structure and morphology of samples were characterized by the SEM (Hitachi S-4800), and AC TEM (Titan G2 60-300 cubed). XRD (Bruker-D8 ADVANCE) was used to investigate the crystal structures. The elemental status was obtained with XPS (Thermo Fisher Scientific). The nitrogen adsorption isotherm was collected with a SI-MP-10 (Quanatachrome). TGA (Pyris 1 DSC) was used to confirm the contents of sulfur in the samples under Ar flow with a heating rate of 10 °C/min. Contact angel was measured on a CA-100C with the sessile drop technique.

Theoretical Calculations:

We have employed the first-principles to perform density functional theory (DFT) calculations within the generalized gradient approximation (GGA) using the Perdew-Burke-Ernzerhof (PBE) formulation. We have chosen the projected augmented wave (PAW) potentials to describe the ionic cores and take valence electrons into account using a plane wave basis set with a kinetic energy cutoff of 450 eV. Partial occupancies of the Kohn-Sham orbitals were allowed using the Gaussian smearing method and a width of 0.05 eV. The electronic energy was considered self-consistent when the energy change was smaller than 10^{-5} eV. A geometry optimization was considered convergent when the energy change was smaller than 0.03 eV $Å^{-1}$. In our structure, the U correction is used for Co atoms. The vacuum spacing in a direction perpendicular to the plane of the structure is 20 Å for the Co_3O_4 and ZnO surfaces. The Brillouin zone integration is performed using 3×3×1 Monkhorst-Pack k-point sampling for a structure. Finally, the adsorption energies

(E_{ads}) were calculated as $E_{ads}= E_{ad/sub} - E_{ad} - E_{sub}$, where $E_{ad/sub}$, E_{ad}, and E_{sub} are the total energies of the optimized adsorbate/substrate system, the adsorbate in the structure, and the clean substrate, respectively. The free energy was calculated using the equation:

$$G=E_{ads}+ZPE-TS$$

where G, E_{ads}, ZPE and TS are the free energy, total energy from DFT calculations, zero point energy and entropic contributions, respectively.

Preparation of Li_2S_6 solution and visualized adsorption tests:

Li_2S and sulfur (1:5, mol%) were mixed and stirred in 1,2-dimethoxyethane (DME) and 1,3-dioxolane (DOL) to obtain Li_2S_6 solution (1 mmol L^{-1}). For polysulfide adsorption tests, 20 mg of the samples were added into 2 mL Li_2S_6 solution and resting for 6 h.

Electrochemical measurements:

Symmetric cells were assembled with two identical electrodes of CZO/HNC, Co_3O_4/HNC, and ZnO/HNC loaded on carbon paper. 0.5 M Li_2S_6 solution in DME/DOL (1:1, vol%) solvents with 1.0 M LiTFSI and 0.2 M $LiNO_3$ was used as electrolyte. CV tests were carried out at scan rates of 10 mV s^{-1} between -0.8 V and 0.8 V. EIS were obtained on an Autolab electrochemical workstation with a frequency range of 100 kHz to 0.1 Hz.

For the Li_2S nucleation tests, the cells were assembled with the CZO/HNC, Co_3O_4/HNC, and ZnO/HNC loaded on carbon paper as working electrodes and metallic Li as counter electrode. 20 μL 1.0 M LiTFSI electrolyte was added into the

anode side and 20 μL 0.5 M Li_2S_8 solution was added into the cathode side. The cells were galvanostatically discharged to 2.06 V at 0.112 mA, then discharged potentiostatically at 2.02 V for 8 h.

For the Li_2S dissolution tests, the cell assembly process is the same as the Li_2S nucleation tests. The cells were galvanostatically discharged to 1.7 V at a constant current of 0.10 mA, then galvanostatically discharged to 1.7 V at 0.01 mA. Last, the cells were potentiostatically charged at 2.35 V for 25 h.

The cathode films were prepared by mixing with 80 wt% S@CZO/HNC, S@Co_3O_4/HNC, or S@ZnO/HNC, 10 wt% KB, and 10 wt% PVDF in NMP, followed with coating on the Al foil and drying in a vacuum oven at 55 °C overnight. The mass loading of S on the electrode is about 1.0-6.7 mg cm^{-2}. Coin cells (CR2032-type) with metallic Li as counter electrode were constructed under an argon-filled glove box using Celgard 2500 as separator and 1.0 M LiTFSI in DME/DOL (1:1, vol%) solvents with 0.2 M $LiNO_3$ as the electrolyte (20 μL mg^{-1} for 1.0-1.5 mg cm^{-2}). The cells were tested in galvanostatic measurements in a voltage range from 1.7 to 2.8 V.

The anode films were prepared by mixing with 90 wt% CZO/HNC and 10 wt% PVDF in NMP, followed with coating on the Cu foil and drying in a vacuum oven at 55 °C overnight. The Li capacity was controlled to be 10 mAh cm^{-2} by galvanostatic charging for 10 h at a current density of 1 mA cm^{-2} used for the subsequent tests (half cell and Li-S full cell). As for the symmetrical cell testing, two identical electrodes with pre-deposition capacity of 10 mAh cm^{-2} (Li@Cu or Li @CZO/HNC) were assembled and cycled. As for the CE measurements, bare Cu or CZO/HNC were used

as the work electrode and Li foil was employed as the counter electrode.

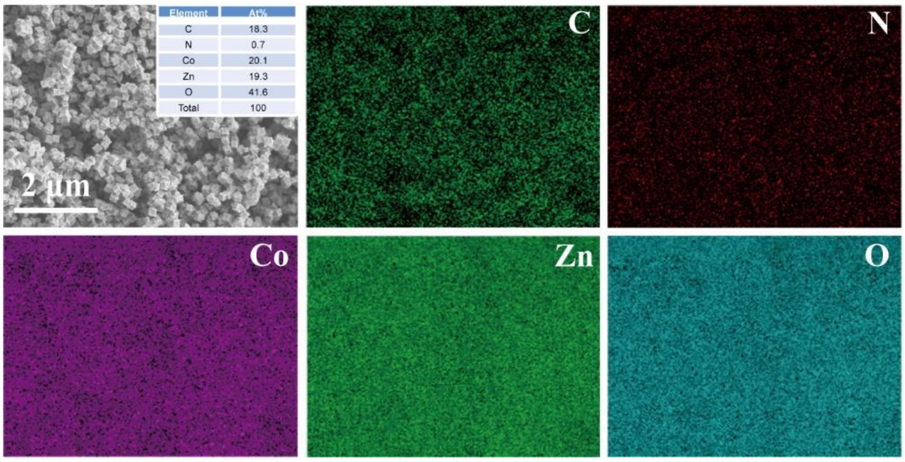

Figure S1. SEM image of CZO/HNC and corresponding element mappings. The inset is the atomic percentages of C, N, Co, Zn, and O elements in CZO/HNC.

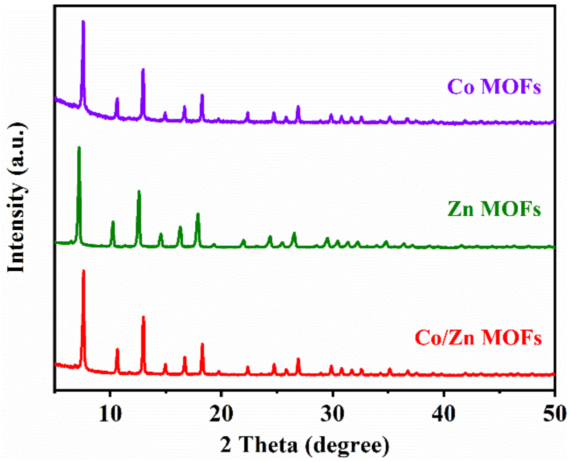

Figure S2. XRD patterns of Co MOFs, Zn MOFs, and Co/Zn MOFs.

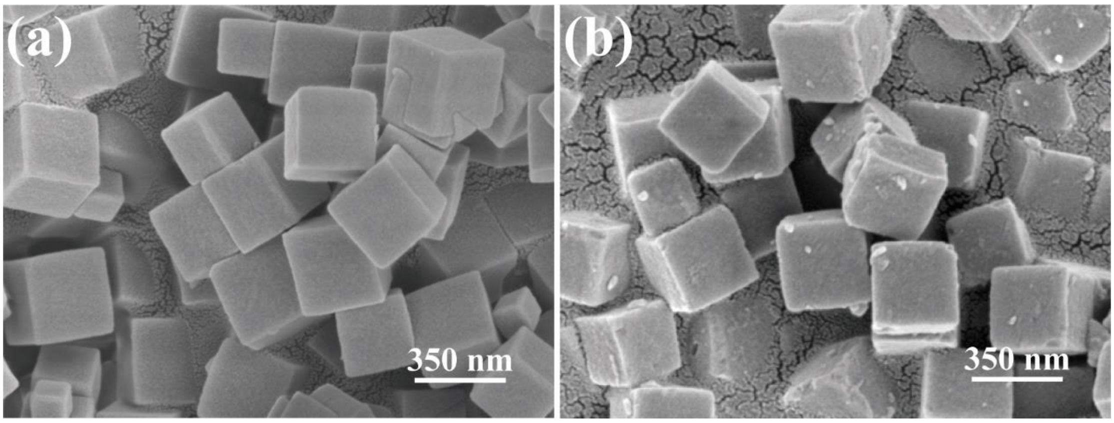

Figure S3. SEM images of (a) Co MOFs and (b) Zn MOFs.

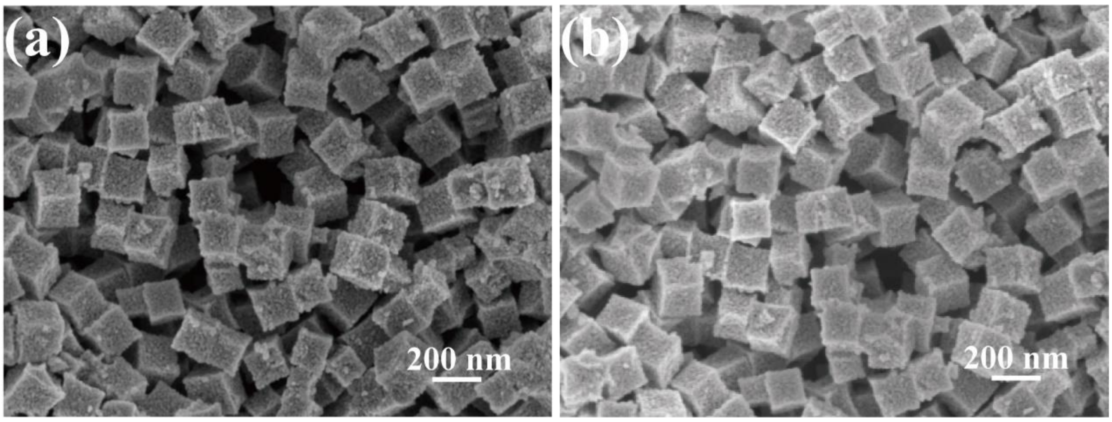

Figure S4. SEM images of (a) Co_3O_4/HNC and (b) ZnO/HNC.

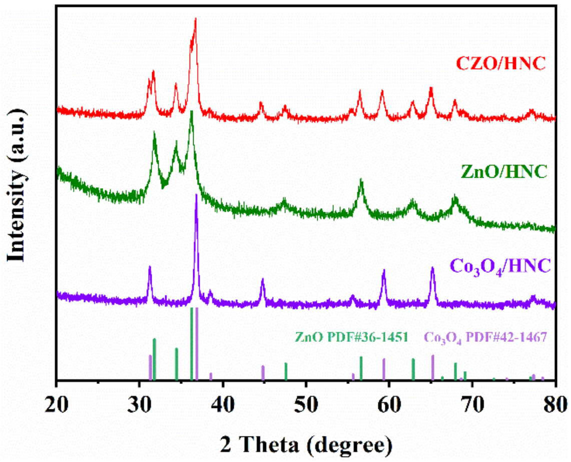

Figure S5. XRD patterns of Co_3O_4/HNC, ZnO/HNC, and CZO/HNC.

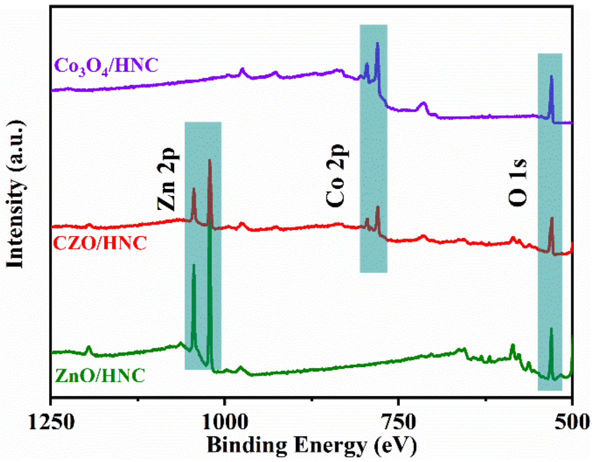

Figure S6. XPS spectra of Co_3O_4/HNC, ZnO/HNC, and CZO/HNC.

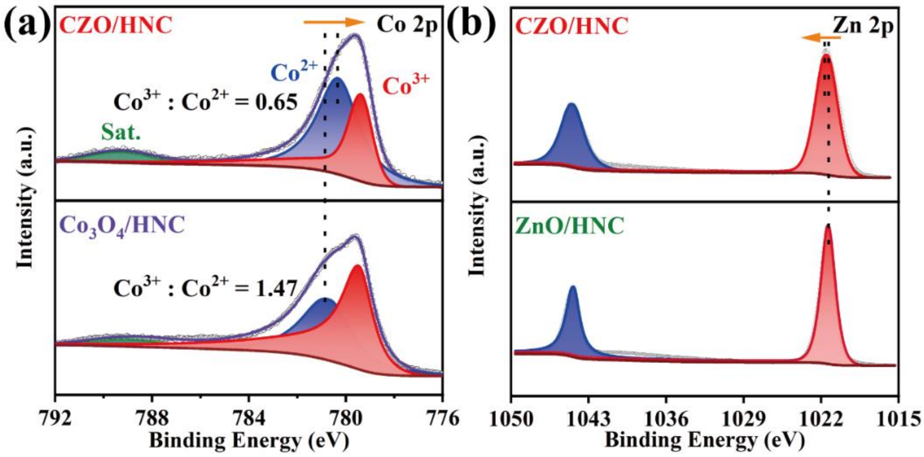

Figure S7. XPS profiles of (a) Co 2*p* and (b) Zn 2*p*.

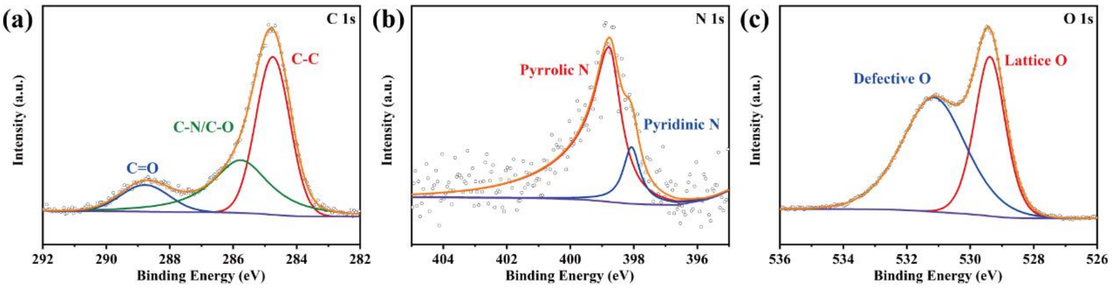

Figure S8. (a) C 1*s*, (b) N 1*s*, and (c) O 1*s* XPS spectra of CZO/HNC.

For CZO/HNC, as shown in Figure S7a, the high-resolution C 1*s* spectra exhibits three peaks observed at 284.8, 285.8, and 287.7 eV, corresponding to C-C, C-N/C-O, and C=O, respectively. The N 1*s* peaks in Figure S7b can be resolved into two peaks centered at 398.1 and 398.8 eV, which can be relative to the pyridinic-N and pyrrolic-N, respectively. Two peaks observed at 529.4 and 531.1 eV in O 1*s* spectra can be indexed to Lattice O and Defective O (Figure S7c).

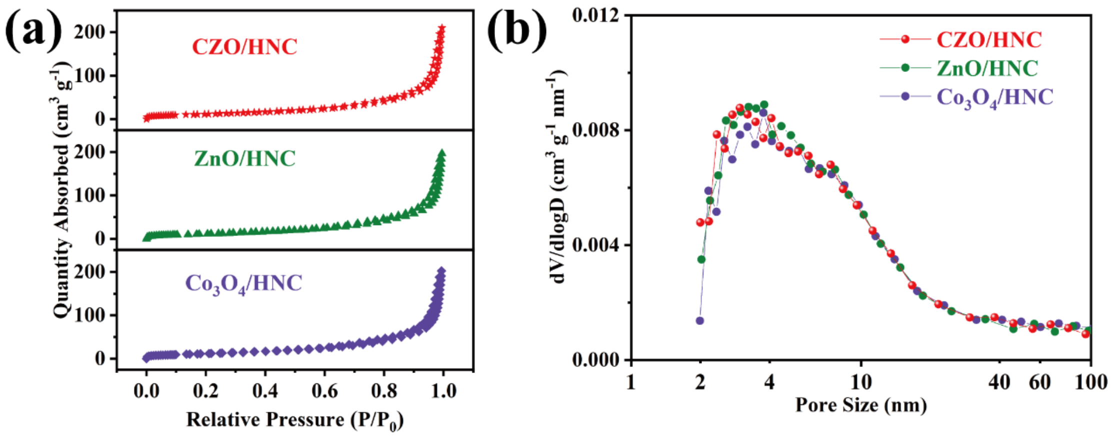

Figure S9. (a) N_2 adsorption-desorption isotherms and (b) pore-size distribution of Co_3O_4/HNC, ZnO/HNC, and CZO/HNC.

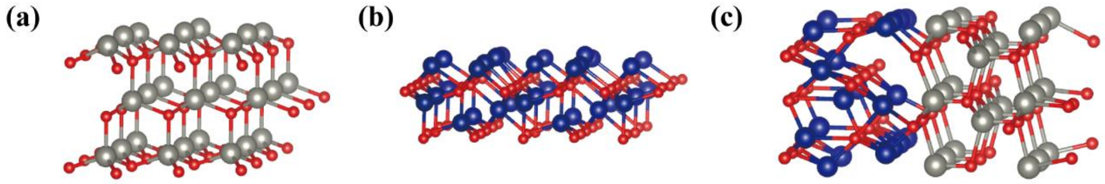

Figure S10. The structures of (a) ZnO, (b) Co_3O_4, and (c) heterointerface of Co_3O_4/ZnO.

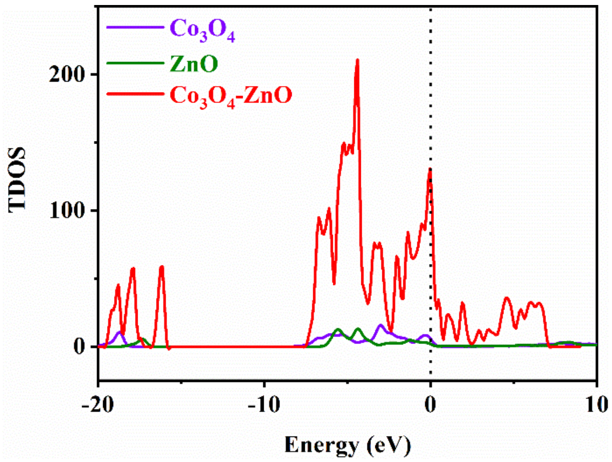

Figure S11. TDOS of ZnO, Co_3O_4, and heterointerface of Co_3O_4/ZnO.

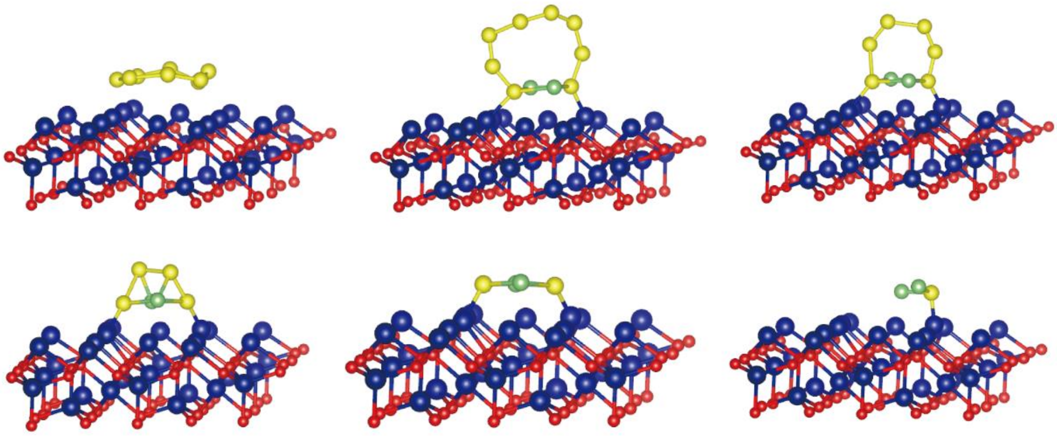

Figure S12. Optimized configurations of sulfur species absorption on Co_3O_4.

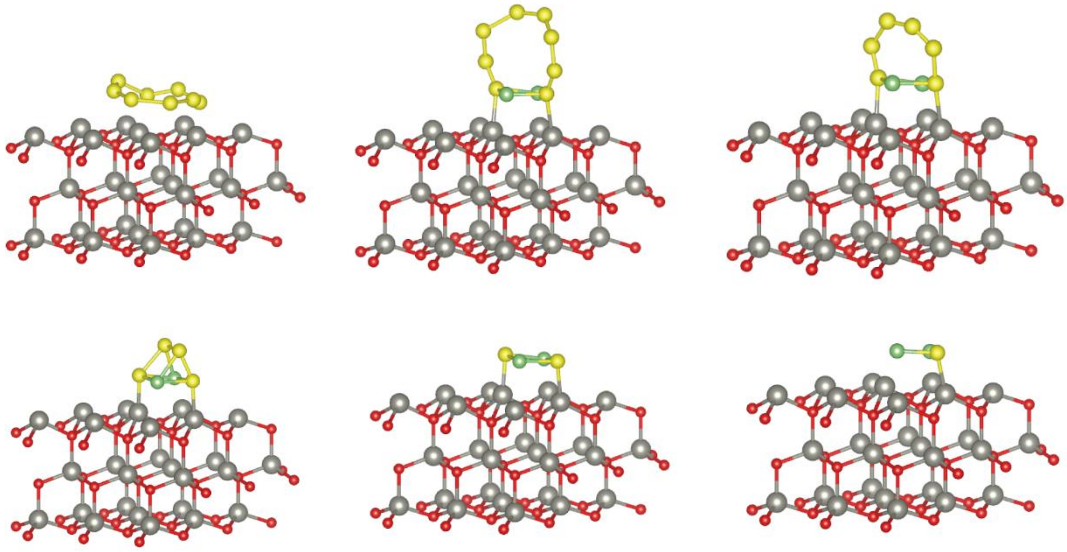

Figure S13. Optimized configurations of sulfur species absorption on ZnO.

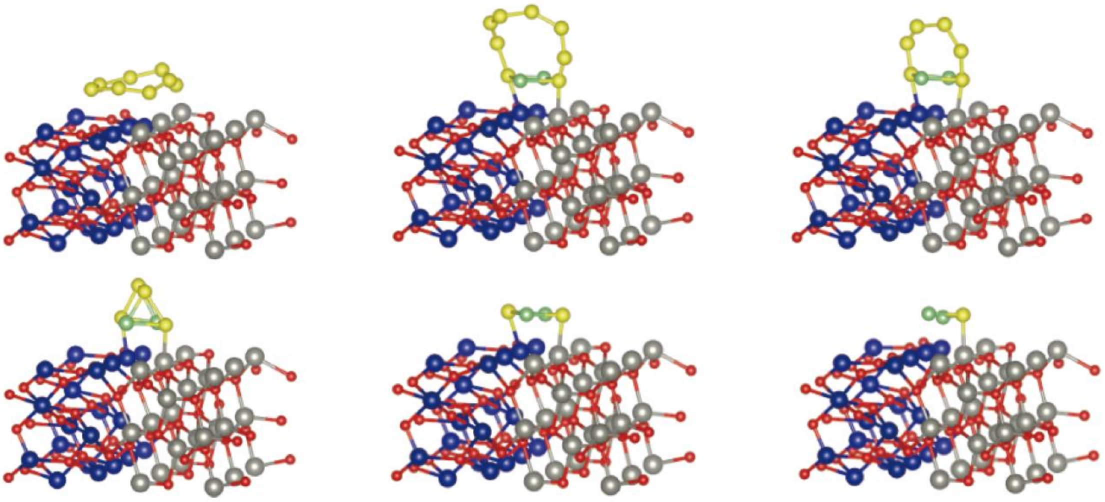

Figure S14. Optimized configurations of sulfur species absorption on Co_3O_4/ZnO.

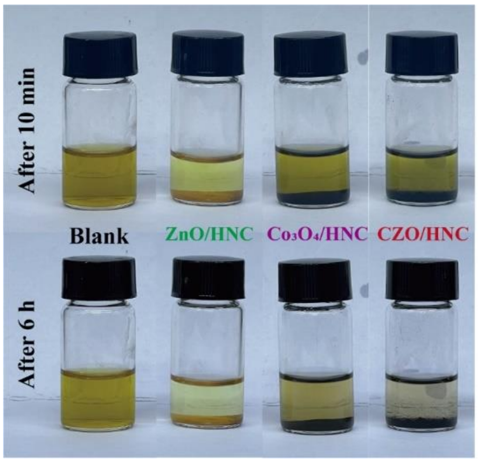

Figure S15. Digital images of Li_2S_6 adsorption test.

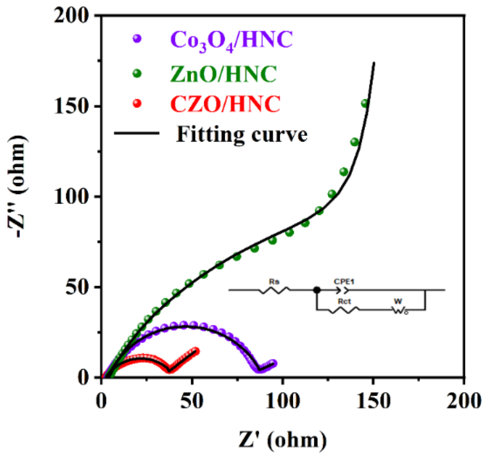

Figure S16. EIS plots of symmetric cells with different electrodes. The inset is the equivalent circuit model.

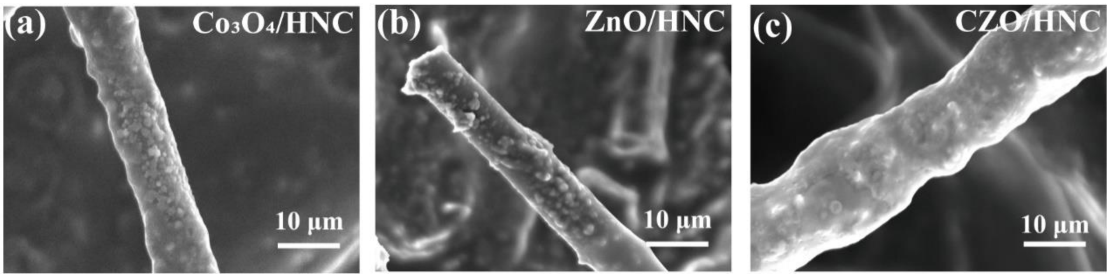

Figure S17. SEM images of different electrodes after potentiostatic discharge.

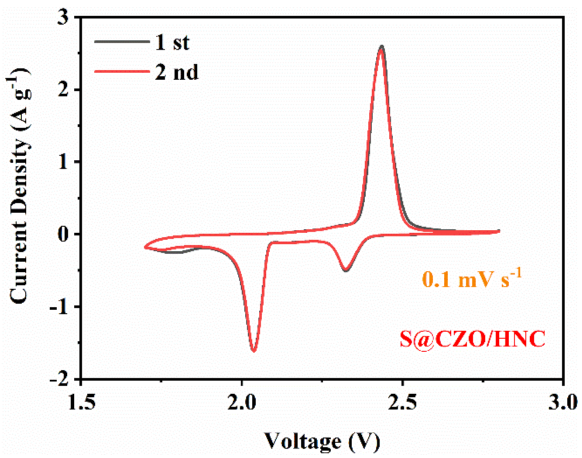

Figure S18. CV profiles of S@CZO/HNC at 0.1 mV s^{-1}.

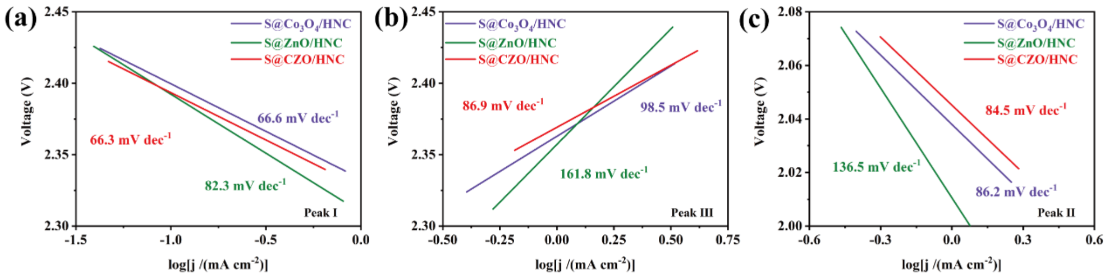

Figure S19. Tafel plots of different cells.

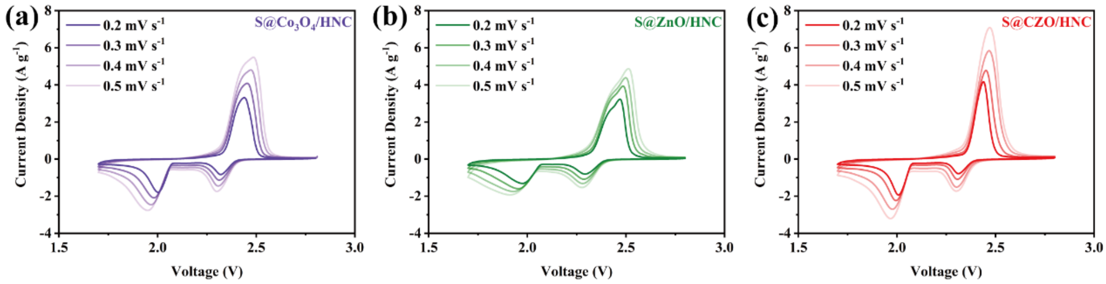

Figure S20. CV curves of different cathodes at different scan rates.

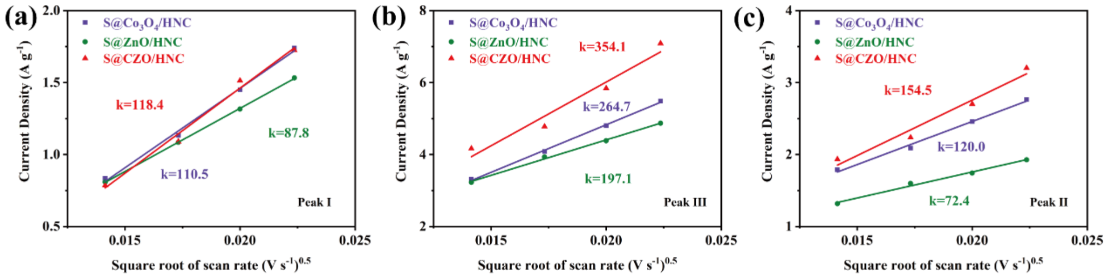

Figure S21. Plot of CV peak current versus square root scan rates for the cells at (a) peak I, (b) peak II, and (c) peak III.

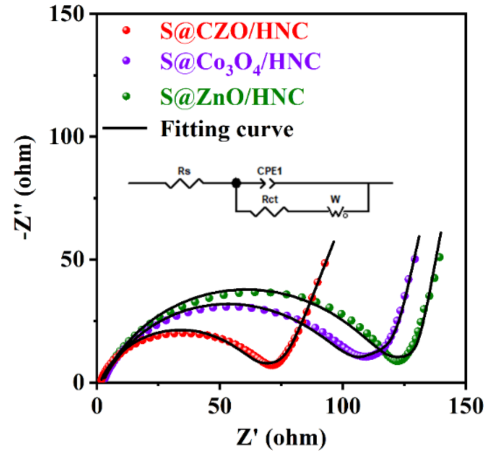

Figure S22. EIS plots of cells with different cathodes. The inset is the equivalent circuit model.

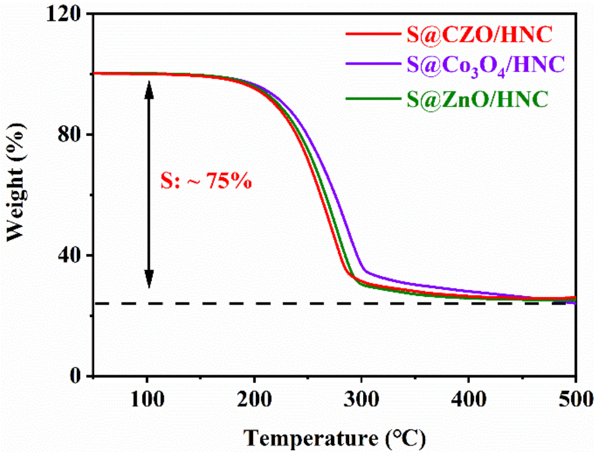

Figure S23. TGA curves of S@CZO/HNC, S@Co_3O_4/HNC and S@ZnO/HNC.

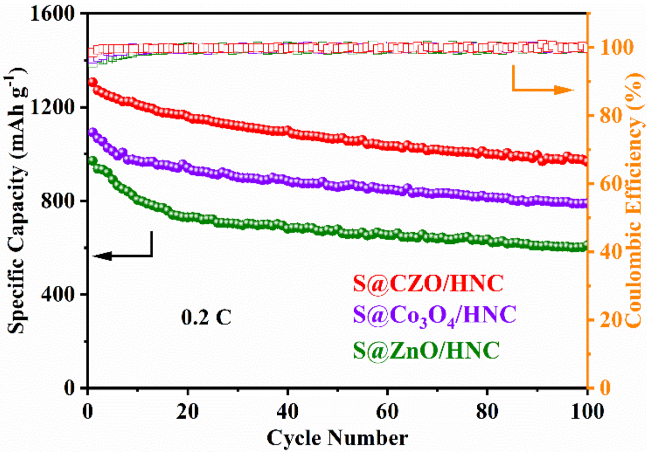

Figure S24. Cycling performance of different cathodes at 0.2 C.

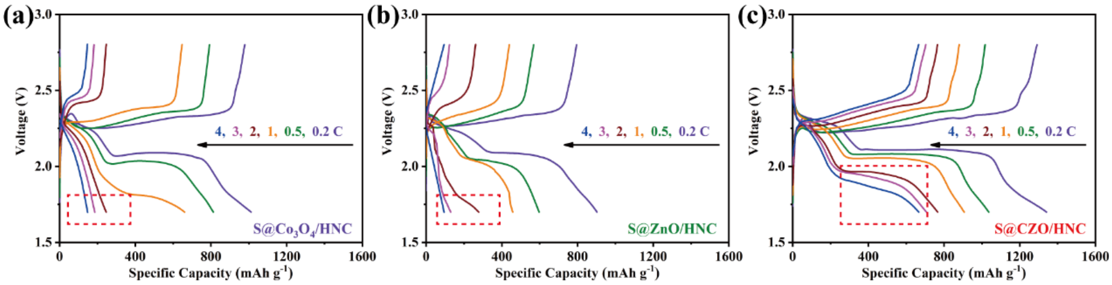

Figure S25. Galvanostatic charge-discharge profiles of three cells at different current density.

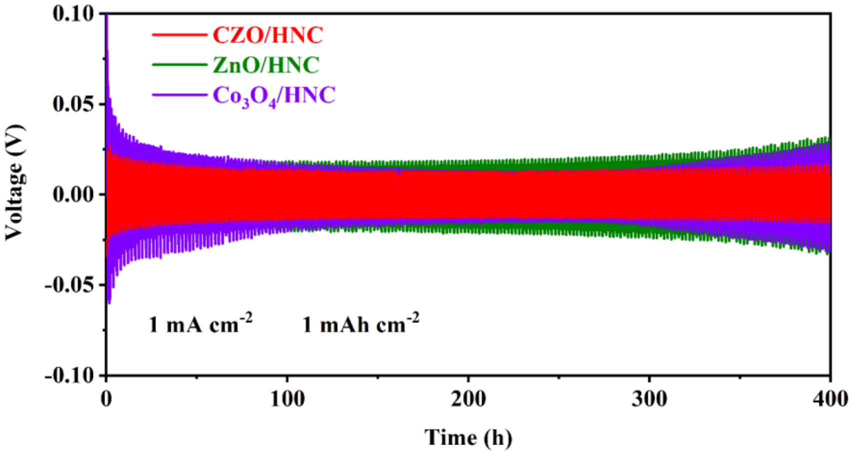

Figure S26. Cycling performance of the symmetrical cells using different electrodes.

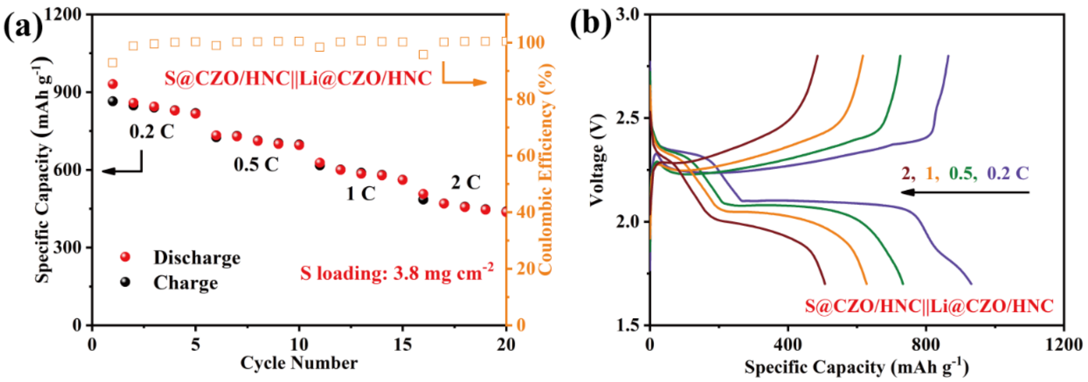

Figure S27. Rate capability of S@CZO/HNC||Li@CZO/HNC full cell with a sulfur loading of 3.8 mg cm^{-2}.

Table S1. Summary of the surface area and pore volume of the three products.

**Sample**	**SSA (m^2 g^{-1})**	**V (m^3 g^{-1})**
Co_3O_4/HNC	66.9	0.33
ZnO/HNC	69.4	0.30
CZO/HNC	69.5	0.31

Table S2. Comparison of electrochemical properties with other dual-functional materials in Li-S full systems.

Sample	Rate (C)	Specific capacity (mAh g^{-1})	Cycle number (*n*)	Capacity decay per cycle (%)	Sulfur loading (mg cm^{-2})	electrolyte content (μL mg^{-1})	Ref.
**CZO/HNC**	2	875.1	1400	0.039	1.2	20	This work
**MnO_x-CeO_2**	1	773.4	1000	0.036	1.5	/	[1]
**TiC@C-TiO_2**	2	754	900	0.065	1.2-1.6	20	[2]
**p-Co_3O_4/n-TiO_2**	1	/	500	0.07	1-1.3	15	[3]
**G@CSOH**	1	/	600	0.068	1	30	[4]
**ZCCDN**	1	809.7	500	0.076	1.8	/	[5]
**eCPAC**	0.5	/	1000	0.053	1.5	/	[6]
**FeSA-PN@PNC**	1	/	800	0.04	1.5	16	[7]
**Ni-CeO_2-CNF**	2	/	1000	0.046	1	/	[8]
**$MoSe_2$@C/rGO**	1	851	300	0.1	1.2-1.5	20	[9]
**nano-Fe_3O_4/PNC**	1	/	800	0.041	1	/	[10]
**CeO_{2-x}@C-rGO**	2	/	500	0.07	1.5	12-15	[11]
**WS_2@NG**	1	/	500	0.087	1.27	25	[12]
**Pt-Nb_2O_5-CNT**	0.5	915	500	0.093	1.3-1.5	25	[13]
**HPPS@SP**	1	/	300	0.09	1	/	[14]
**RuO_{2-x}**	1	1157.6	600	0.08	/	/	[15]

**CNC Ni-Pt/G**	1	591.9	1000	0.025	1.3	15	[16]
**VPC**	2	874.7	600	0.076	/	15	[17]
**VO_2-rGO**	0.5	/	700	0.065	1.2-1.8	/	[18]
**Co-V_2O_5-YS**	0.5	/	300	0.11	1.2	/	[19]

References

[1] Z. Kong, Y. Li, Y. Wang, Y. Zhang, K. Shen, X. Chu, H. Wang, J. Wang, L. Zhan, *Chem. Eng. J.* **2020**, 392, 123697.

[2] X. Zhang, W. Yuan, Y. Yang, Y. Chen, Z. Tang, C. Wang, Y. Yuan, Y. Ye, Y. Wu, Y. Tang, *Small* **2020**, 16, 2005998.

[3] H. Li, C. Chen, Y. Yan, T. Yan, C. Cheng, D. Sun, L. Zhang, *Adv. Mater.* **2021**, 35, 2105067.

[4] X. Wang, J. Han, C. Luo, B. Zhang, J. Ma, Z. Li, Y. B. He, Q. H. Yang, F. Kang, W. Lv, *Small* **2021**, 17, 2101538.

[5] P. Xu, H. Liu, Q. Zeng, X. Li, Q. Li, K. Pei, Y. Zhang, X. Yu, J. Zhang, X. Qian, R. Che, *Small* **2021**, 17, 2005227.

[6] Y. Cui, J. Li, Y. Cai, H. Zhang, S. Zhang, *Small* **2022**, 18, 2204183.

[7] T. Huang, Y. Sun, J. Wu, Z. Shi, Y. Ding, M. Wang, C. Su, Y. y. Li, J. Sun, *Adv. Func. Mater.* **2022**, 32, 2203902.

[8] Y. Kong, X. Ao, X. Huang, J. Bai, S. Zhao, J. Zhang, B. Tian, *Adv. Sci.* **2022**, 9, 2105538.

[9] C. Li, W. Ge, S. Qi, L. Zhu, R. Huang, M. Zhao, Y. Qian, L. Xu, *Adv. Energy Mater.*

2022, 2103915.

[10] M. Li, S. Ji, X. Ma, H. Wang, X. Wang, V. Linkov, R. Wang, *ACS Appl. Mater. Interfaces* **2022**, 14, 16310.

[11] Y. Li, X. Zhang, Q. Zhang, J. Cui, X. Liang, J. Yan, J. Liu, H. H. Tan, Y. Yu, Y. Wu, *ACS Appl. Mater. Interfaces* **2022**, 14, 18634.

[12] X. Liu, H. Rao, K. Sun, H. Gou, T. Lu, Y. Qian, *Appl. Surf. Sci.* **2022**, 599, 154022.

[13] Y. Liu, D. Hong, M. Chen, Z. Su, Y. Gao, Y. Zhang, D. Long, *Chem.Eng. J.* **2022**, 430, 132714.

[14] Z. Luo, S. Tao, Y. Tian, H. Tu, L. Xu, W. Deng, G. Zou, H. Hou, X. Ji, *Nano Res.* **2022**, DOI: 10.1007/s12274-022-5029-4.

[15] L. Niu, T. Wu, D. Zhou, J. Qi, Z. Xiao, *Energy Storage Mater.* **2022**, 45, 840.

[16] Z. Y. Wang, B. Zhang, S. Liu, G. R. Li, T. Yan, X. P. Gao, *Adv. Func. Mater.* **2022**, 2200893.

[17] Q. Yang, Z. Chen, Z. Zheng, L. Chen, L. Song, J. Sun, Y. Song, *Chem. Commun.* **2022**, 58, 5347.

[18] B. Chen, J. Wei, X. Li, Y. Ji, D. Liang, T. Chen, *J. Colloid Interface Sci.* **2023**, 629, 1003.

[19] F.-J. Liu, W.-L. Luo, Z. Zhang, J. Yu, J.-X. Cai, Z.-Y. Yang, *Chem. Eng. J.* **2023**, 456, 140948.
